# Discovery of the Fe-analogue of akimotoite in the shocked Suizhou L6 chondrite

**DOI:** 10.1038/srep42674

**Published:** 2017-02-15

**Authors:** Luca Bindi, Ming Chen, Xiande Xie

**Affiliations:** 1 Dipartimento di Scienze della Terra, Università di Firenze, Via La Pira 4, I-50121 Florence, Italy; 2 CNR-Istituto di Geoscienze e Georisorse, Via La Pira 4, I-50121 Florence, Italy; 3 State Key Laboratory of Isotope Geochemistry, Guangzhou Institute of Geochemistry, Chinese Academy of Sciences, Guangzhou 510640, China; 4 Guangdong Provincial Key Laboratory of Mineral Physics and Materials, Guangzhou 510640, China; 5 Key Laboratory of Mineralogy and Metallogeny, Guangzhou Institute of Geochemistry, Chinese Academy of Sciences, Guangzhou 510640, China

## Abstract

We report the first natural occurrence of the Fe-analogue of akimotoite, ilmenite-structured MgSiO_3_, a missing phase among the predicted high-pressure polymorphs of Fe-pyroxene, with the composition (Fe^2+^_0.48_Mg_0.37_Ca_0.04_Na_0.04_Mn^2+^_0.03_Al_0.03_Cr^3+^_0.01_)_Σ=1.00_Si_1.00_O_3_. The new mineral was approved by the International Mineralogical Association (IMA 2016-085) and named hemleyite in honour of Russell J. Hemley. It was discovered in an unmelted portion of the heavily shocked L6 Suizhou chondrite closely associated to olivine, clinoenstatite and Fe-bearing pyroxene with a composition nearly identical to that of hemleyite. We also report the first single-crystal X-ray diffraction study of a Si-bearing, ilmenite-structured phase. The fact that hemleyite formed in a meteorite exposed to high pressures (<20 GPa) and temperatures (<2000 °C) during impact-induced shocks indicates that it could play a crucial role at the bottom of the Earth’s mantle transition zone and within the uppermost lower mantle.

The mineralogy of Earth’s deep interior represents a fascinating challenge for geoscientists. Key information can be obtained from the study of mantle xenoliths^1^ and inclusions in diamonds^2^,^3^, as well as from experimental studies of phase equilibria of silicates and oxides^4^,^5^. However, most of the major Earth and rocky planet-forming materials [i.e., *M*-Si-oxides (*M* = Mg, Fe)] such as majorite^6^, akimotoite^7^,^8^, wadsleyite^9^, ringwoodite-ahrensite^10^,^11^, and bridgmanite^7^,^12^, have been discovered in shocked meteorites. Shocked meteorites are extraterrestrial rocks that have experienced high-pressure and high-temperature collisions in outer space, fundamental processes affecting planets and asteroids through the evolution of the solar system. As a direct product of these impact events, shock metamorphism in meteorites provides many dense silicate minerals that are thought to compose Earth’s transition zone and lower mantle. Among them, akimotoite, MgSiO_3_ with ilmenite structure, is thought to exist at the bottom of the Earth’s mantle transition zone and within the uppermost lower mantle, especially under low-temperature conditions^13^,^14^. Akimotoite is considered a major constituent of the harzburgite layer of subducting slabs, and the most anisotropic mineral in the mantle transition zone^15^,^16^,^17^. The garnet-akimotoite and akimotoite-bridgmanite phase transitions are associated with steep density and sound velocity increases and may be responsible for the multiple seismic discontinuities near the 660 km depth^13^,^18^. Due to the fact that no ilmenite or the orthorhombic perovskite modifications of FeSiO_3_ had been found in experiments^19^, most of the studies have focused on MgSiO_3_ akimotoite, without considering the influence of iron on its stability and density. Only recently, it was demonstrated that Fe^3+^-rich akimotoite is stable for a wider range of pressures than MgSiO_3_ akimotoite and that it may play an important role in subduction regions such as the Pacific plate beneath southern California^20^. Furthermore, it has been shown that Fe^2+^-akimotoite could derive from a progressive transformation of clinoferrosilite at pressure higher than 36 GPa^21^.

Here we report the discovery of the first natural occurrence of the Fe-analogue of akimotoite, with ideal formula FeSiO_3_. The new mineral was found in an unmelted portion of the Suizhou meteorite, and named after Russell J. Hemley (b. 1954) former Director of the Geophysical Laboratory of the Carnegie Institution of Washington D.C., USA, and very well-known in the scientific community for his research exploring the behavior of materials under extreme conditions of pressure and temperature. The new mineral and mineral name have been approved by the Commission on New Minerals, Nomenclature and Classification of the International Mineralogical Association (IMA 2016-085). Holotype material is deposited in the collections of the Museo di Storia Naturale, Università degli Studi di Firenze, Via La Pira 4, I-50121, Firenze, Italy, catalogue number 3238/I.

## Results and Discussion

### The Suizhou meteorite

This meteorite fell on April 15, 1986, in Dayanpo, which is located 12.5 km southeast of Suizhou in Hubei, China. A total of 270 kg of the Suizhou meteorite was collected, and the largest fragment, weighting 56 kg, is now preserved in the City Museum of Suizhou. This meteorite was classified as a shock-metamorphosed L6-chondrite^22^, with an estimated shock stage S5 according to the classification of shock metamorphism^23^. The meteorite contains shock-produced melt veins less than three hundred micrometers in thickness. The shock veins contains abundant high-pressure polymorphs including ringwoodite, majorite, majorite-pyrope garnet, akimotoite, magnesiowüstite, lingunite, tuite, xieite^24^,^25^,^26^,^27^,^28^,^29^,^30^,^31^,^32^. A (Mg,Fe)SiO_3_-glass was also identified in the shock veins, which was suggested to be possibly a vitrified perovskite^30^.

### Description of the sample

**Figure 1:**
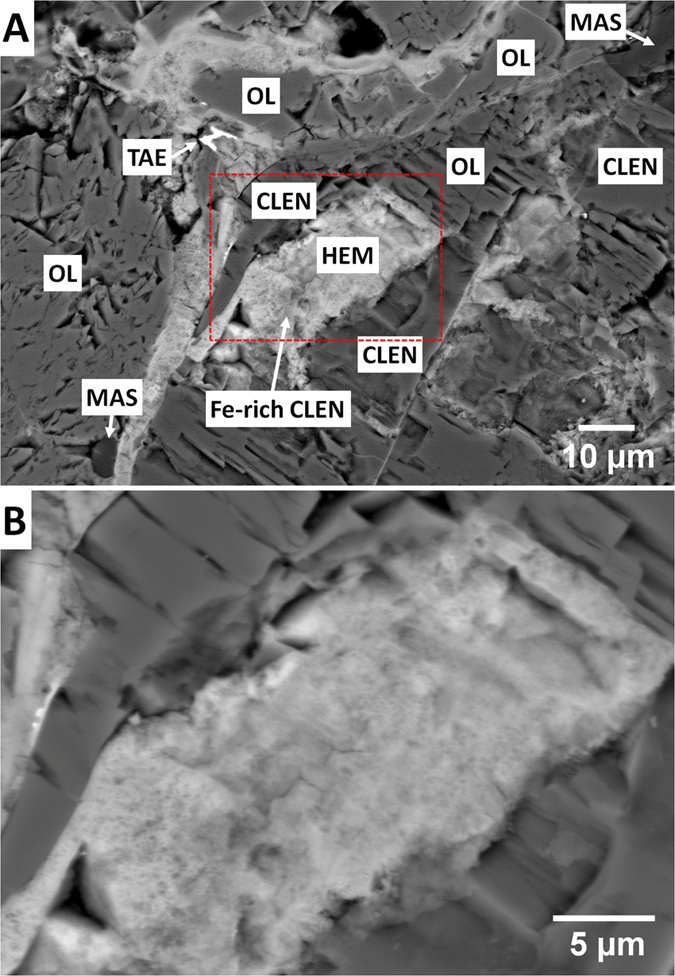
Backscattered electron images of a section of the Suizhou meteorite. (**A**) Section of the meteorite with labeling of the phases (HEM = hemleyite; CLEN = clinoenstatite; OL = olivine; MAS = maskelynite; TAE = taenite); red dashed box indicates the region to be enlarged in (**B**). (**B**) The hemleyite grain used for the Raman and the X-ray structural study.

The new mineral hemleyite occurs as one subhedral crystal, about 7 × 6 × 5 μm in size, coexisting with Fe-rich clinoenstatite (which is mineralogically a clinoferrosilite but we will refer at it as Fe-rich clinoenstatite or Fe-pyroxene) and closely associated to Fe-poor clinoenstatite and forsteritic olivine (Fig. 1). The chemical composition of these phases is given in Table 1. Color, lustre, streak, hardness, tenacity, cleavage, fracture, density, and optical properties could not be determined because of the small grain size. The calculated density using the empirical formula and X-ray single-crystal data is 4.383 g·cm^−3^.

**Figure 2:**
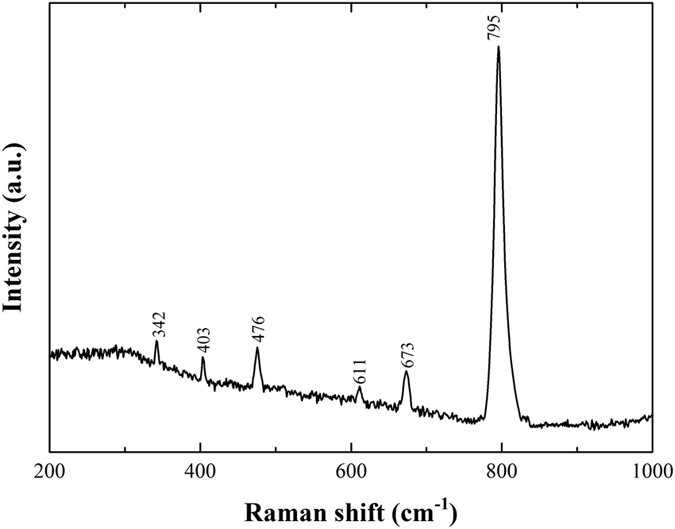
Raman spectrum of hemleyite.

Hemleyite was initially identified by Raman spectroscopy. The Raman spectrum of hemleyite (Fig. 2) displays bands at 795, 673, 611, 476, 403 and 342 cm^−1^ with the typical strong peak at 795 cm^−1^, corresponding to the stretching vibrations of the SiO_6_ octahedra. The hemleyite Raman spectrum is similar to that obtained for akimotoite from the Suizhou meteorite^32^ and those for akimotoite from other L-group chondrites^33^,^34^,^35^,^36^. As already observed for akimotoite from Suizhou^32^, Raman bands of hemleyite are much sharper than those observed for other ilmenite-type polymorphs in other chondrites, thus indicating rather high crystallinity.

To get more information on the crystal structure and the relationships with the surrounding minerals, the small hemleyite fragment was handpicked from the polished section under a reflected light microscope from the upper part of the grain in Fig. 1B and mounted on a 5 μm diameter carbon fiber, which was, in turn, attached to a glass rod. Then, the fragment was tested by single-crystal X-ray diffraction and was found to consist of crystalline hemleyite (single-crystal spots in Figure S1) associated to minor, fine-grained polycrystalline pyroxene (diffraction rings in Figure S1).

### Crystal structure of hemleyite

**Figure 3:**
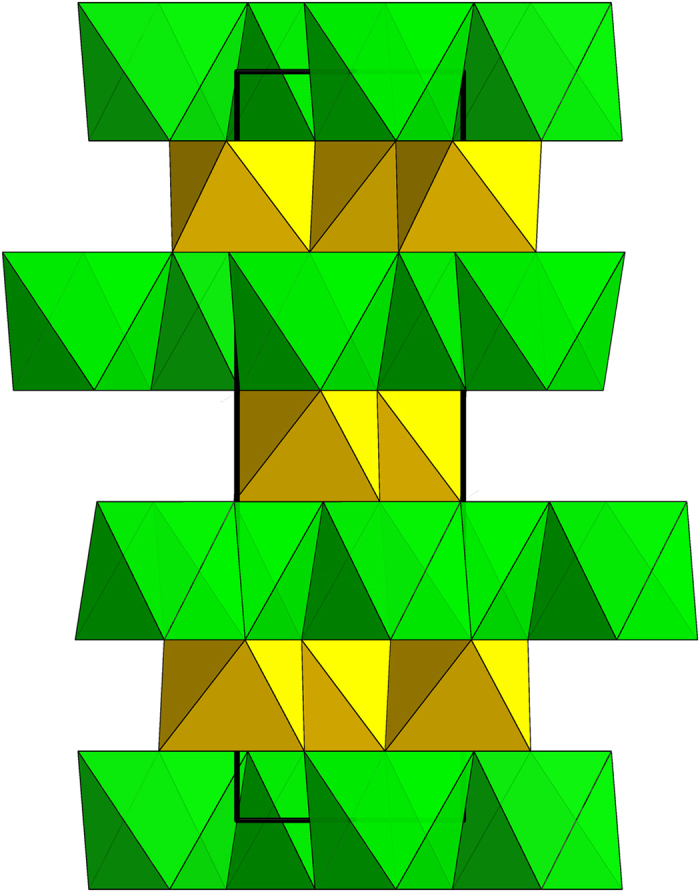
The crystal structure of hemleyite projected down [010]. The vertical direction represents the **c**-axis. (Fe,Mg)- and Si-octahedra are depicted in green and yellow, respectively. The unit-cell is outlined.

The crystal structure of hemleyite is shown in Fig. 3. It consists of a lattice of hexagonal close-packed O atoms in which only two-thirds of the octahedral sites are occupied. The octahedra share edges to form 6-membered rings, thus forming sheets parallel to (0001). The sheets are linked into a framework by sharing faces and corners of octahedra. In hemleyite, the presence of (Fe,Mg) and Si specifically ordered into two octahedral sites (i.e., A and B) causes a decrease in symmetry from the corundum-type structure, space group *R*-3*c*, to *R*-3. Final atomic coordinates and equivalent isotropic displacement parameters are given in Table S1, anisotropic displacement parameters in Table S2, whereas selected bond distances are shown in Table S3.

The A site showed a mean electron number of 18.7 and the B site 14.0. The latter was thought to be fully occupied by silicon. The site population inferred at the larger A octahedral position (Fe^2+^_0.48_Mg_0.37_Ca_0.04_Na_0.04_Mn^2+^_0.03_Al_0.03_Cr^3+^_0.01_) gives a mean electron number of 19.6, in good agreement with the site scattering observed at this site. Moreover, taking into account the weighted sum of ionic radii^37^, we get a mean ideal bond value of 2.14 Å, close to the observed value from the structure refinement (<A-O> = 2.13 Å; Table S3). These crystal-chemical considerations, together with the perfect charge balance of the formula, point to the presence of Fe (and Mn) and Cr in the divalent and trivalent states, respectively.

**Figure 4:**
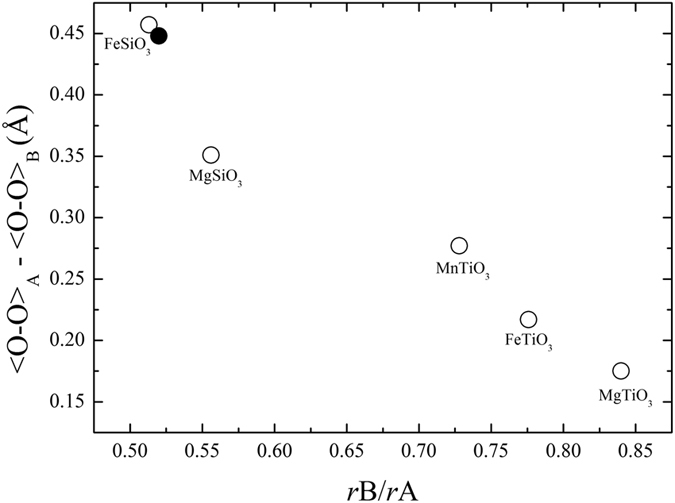
Structural details of hemleyite. The difference between the O-O edge of the A and B sites (in Angstrom), which is directed along the **c**-axis, plotted as a function of the ratio between the ionic radius of the B and A cation. Filled circle corresponds to hemleyite (this study), whereas empty circles refer to literature data as follows: FeSiO_3_^41^, MgSiO_3_^38^, MnTiO_3_^59^, FeTiO_3_^60^, MgTiO_3_^61^.

The unit-cell parameters of hemleyite are strongly influenced by the entry of Fe into the structure. We observed a general expansion of the unit cell from pure MgSiO_3_^38^. The assignment of Fe substituting for Mg at the A site is required both to account for the electron density at that site and to justify the increase of the mean bond distance (2.13 Å) relative to pure MgSiO_3_ (2.077 Å; Horiuchi *et al*.^38^). Similar to what happens in other Mg-Fe silicates^39^, the Fe–for–Mg substitution does not induce a distortion of the A-octahedral site (σ^2^ = 140.3 and 143.4 in hemleyite and pure MgSiO_3_, respectively). On the other hand, the Fe–for–Mg substitution has effects on the neighbouring Si-bearing B-octahedral site, with an increase of σ^2^ (Robinson *et al*.^40^) from 52.8 in pure MgSiO_3_^38^ to 63.9 in hemleyite. If we calculate the difference between the O–O edge of the A and B sites, which is directed along the **c**-axis, and plot it as a function of the ratio between the ionic radius of the B and A cations (Fig. 4), an almost linear trend can be observed, with hemleyite falling close to ideal FeSiO_3_^41^.

### Crystal-chemical considerations

**Figure 5:**
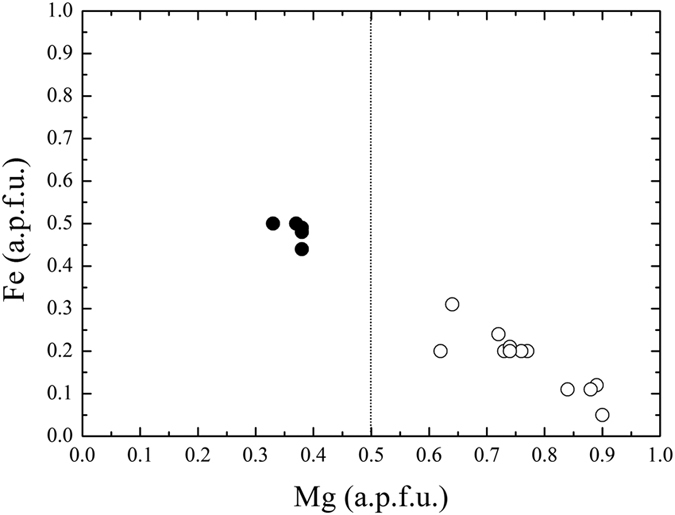
Fe versus Mg in akimotoite. Mg contents (in a.p.f.u., atoms per formula unit) plotted against the Fe contents (in a.p.f.u) for akimotoites. Filled circles correspond to hemleyite, empty circles refer to literature data^8^,^32^,^33^,^35^,^42^,^43^,^44^,^45^,^46^,^47^,^48^,^49^. The dashed line indicates the 50%-limit.

If we plot the Fe and Mg contents of all the akimotoites reported in literature^8^,^32^,^33^,^35^,^42^,^43^,^44^,^45^,^46^,^47^,^48^,^49^, we can observe that a discrete variation is present, with only the phase from the Suizhou meteorite described here crossing the boundary delimiting akimotoite and hemleyite (dashed line in Fig. 5). Although the chemical composition of hemleyite is far from the ideal FeSiO_3_ end member, Fe is the dominant cation at the A site of the ilmenite-type structure. Furthermore, we always found Fe > Mg in all the microprobe analyses carried out on the grain depicted in Fig. 1B (Table 1) and used for the structural investigation.

**Figure 6:**
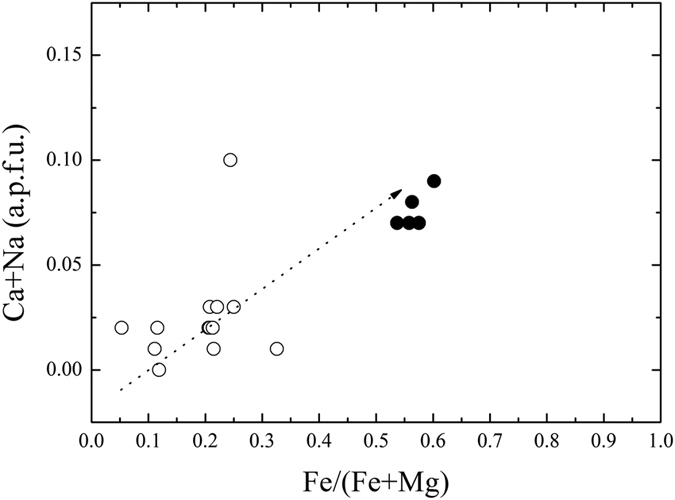
Chemical characteristics of akimotoite/hemleyite. Fe/(Fe + Mg) ratios plotted against the (Ca + Na) contents (in a.p.f.u) for the akimotoite/hemleyite solid solution. Filled circles correspond to hemleyite, empty circles refer to literature data^8^,^32^,^33^,^35^,^42^,^43^,^44^,^45^,^46^,^47^,^48^,^49^. The dashed arrow gives the tendency.

Another interesting feature is related to the minor (Ca + Na) amounts in the ilmenite structure. Minor contents of these elements were detected in all the reported akimotoite occurrences^8^,^32^,^33^,^35^,^42^,^43^,^44^,^45^,^46^,^47^,^48^,^49^. With increasing the Fe/(Fe + Mg) ratio, an increase of the (Ca + Na) content in akimotoite is evident (Fig. 6). The only value going off the main trend is the akimotoite from the Tenham meteorite studied by Ferroir *et al*.^35^. The presence of Fe^2+^replacing Mg induces an increase of the size of the A octahedra and, more generally, an overall enlargement of the ilmenite-structure topology. Such an enlargement favors the entry of larger cations, such as Ca and Na.

### Formation hypothesis for hemleyite

Static high-pressure experiments demonstrated that low-Ca pyroxene could transform to akimotoite at 17.5–27.5 GPa and 600–2050 °C^19^,^50^. The transformation of enstatite to akimotoite requires temperatures in excess of 1550 °C at 22 GPa^51^. Two scenarios or models were proposed for the formation mechanisms of akimotoite in shock-metamorphosed meteorites: (1) solid state transformation from low-Ca pyroxene or clinopyroxene to akimotoite under high pressures and temperatures^7^,^8^; (2) crystallization from a chondritic silicate melt under high pressures and temperature^42^. The first model is mainly based on a topotaxial relationship between akimotoite with neighboring pyroxene, and the same composition between pyroxene and akimotoite^7^,^8^, and the second one is because akimotoite is enriched in CaO, Al_2_O_3_ and Na_2_O with respect to the coexisting pyroxene^42^. In the Suizhou L6 chondrite, hemleyite occurs mainly along the cracks within silicates (Mg-rich pyroxene and Mg-rich olivine). The hemleyite fragment we have studied (Fig. 1B) was found to intimately coexist with Fe-rich clinoenstatite and be surrounded by Fe-poor clinoenstatite. Such petrographic evidence could lead to the following formation mechanism: (1) Fe-rich clinoenstatite belongs to a kind of secondary pyroxene likely formed from olivine and pyroxene by replacement of Fe-rich materials during the thermal metamorphism of the meteorite, and (2) a later impact-induced shock transformed some Fe-rich clinoenstatite into hemleyite. The pressure and temperature conditions (18–20 GPa and 1800–2000 °C) of shock-veins in the Suizhou meteorite^32^ can be estimated according to the liquidus phases of high-pressure minerals. However, the *P*–*T* conditions for the formation of hemleyite in the chondritic portion cannot be estimated because of the lack of available phase diagram for the solid-state crystallization of ilmenite-structured (Fe,Mg)SiO_3_.

### Implications

Neither Fe-dominant MgSiO_3_ nor pure FeSiO_3_ with ilmenite structure have been synthesized yet. Indeed, high-pressure experiments^52^ in the system MgSiO_3_-FeSiO_3_ at about 23 GPa at 1100 °C show that ilmenite cannot contain more than 15 mol% of FeSiO_3_. For FeSiO_3_-richer compositions, the experimental products are ilmenite s.s. (FeSiO_3_) + ringwoodite s.s (Mg_2_SiO_4_) + stishovite (SiO_2_). We think that the difference between the experimental results^52^ and what shown in the present study is not linked to the extremely rapid cooling rates occurring in impact-induced shocks, which can allow to discover phases that would not be experimentally recovered under ambient conditions. The most likely reasons could be (*i*) a metastable transition of Fe-rich pyroxene to hemleyite, or (*ii*) a temperature-dependent increase of solubility of the FeSiO_3_ component in the ilmenite structure. The first hypothesis is that inferred to explain the transition of Na-rich hollandite (lingunite) from feldspar^53^. The second, which seems more unlikely, cannot be properly weighted at present as experimental data are still insufficient to evaluate how much FeSiO_3_ component is dissolved in ilmenite in the range of temperature 1800–2000 °C.

The discovery of the Fe-dominant akimotoite, i.e. hemleyite, is important not only to shed further light on asteroidal impact events but also for mantle geophysics as it expands the knowledge of the most important mantle minerals. To know that also ilmenite-structured MgSiO_3_ can contain high Fe contents is important to study the effect of this chemical substitution on the acoustic velocity and density profiles in high-pressure models and for a comparison with seismically derived Earth velocity and density structure obtained with computational approaches^54^. The ideas of multiple seismic discontinuities near the 660 km boundary in the subduction zone^55^, instead of a single, sharp velocity jump at the lower-mantle boundary, strongly suggest the presence of akimotoite/hemleyite solid solution at this depth in the peridotitic lithology of subducted slabs.

## Methods

### Studied material

A small piece of the Suizhou meteorite (about 10 g), was sliced in 6 different pieces. Each piece was embedded in epoxy, polished with diamond pastes, and then studied by scanning electron microscopy and electron microprobe.

### Scanning electron microscopy

The instrument used was a Zeiss - EVO MA15 Scanning Electron Microscope coupled with an Oxford INCA250 energy-dispersive spectrometer, operating at 25 kV accelerating potential, 500 pA probe current, 2,500 cps as average count rate on the whole spectrum, and a counting time of 500 s. Samples were sputter-coated with 30-nm-thick carbon film.

### Electron microprobe

Quantitative analyses on the different phases were carried out using a JEOL JXA 8600 microprobe (WDS mode, 15 kV, 10 nA, 1 μm beam size, counting times 20 s for peak and 10 s for background). High spatial resolution was achieved using conditions of 13 kV, 7 nA. For the WDS analyses the *K*α lines for all the elements were used. The standards employed were: albite (Na, Al, Si), synthetic Cr_2_O_3_ (Cr), ilmenite (Fe), olivine (Mg), diopside (Ca), and bustamite (Mn).

### Single-crystal X-ray diffraction and structure refinement

Single-crystal X-ray studies were carried out using a Oxford Diffraction Xcalibur 3 diffractometer equipped with an Oxford Diffraction CCD detector, with graphite-monochromatized Mo*K*α radiation (λ = 0.71073 Å), working conditions 50 kV × 50 nA and with 300 s exposure time per frame; the detector-to-sample distance was 6 cm. Hemleyite is trigonal, space group *R*-3, with unit-cell parameters (hexagonal setting): *a* = 4.7483(5), *c* = 13.665(1) Å, *V* = 266.82(6) Å^3^, *c*:*a* = 2.8779, Z = 6.

Single-crystal X-ray diffraction intensity data of hemleyite were integrated and corrected for standard Lorentz and polarization factors with the *CrysAlis* RED package^56^. The program ABSPACK in *CrysAlis* RED^56^ was used for the absorption correction. A total of 270 unique reflections was collected. Given the similarity in the unit-cell values and in the space groups, the structure was refined starting from the atomic coordinates reported for akimotoite^37^ using the program Shelxl-97^57^. The site occupation factor (s.o.f.) at the cation sites was allowed to vary (Fe vs. Mg and Si vs. structural vacancy for the A and B sites, respectively) using scattering curves for neutral atoms taken from the *International Tables for Crystallography*^58^. In terms of mean electron number, the X-ray formula, (Fe_0.48_Mg_0.52_)SiO_3_, is in excellent agreement with that obtained with the electron microprobe, (Fe^2+^_0.48_Mg_0.37_Ca_0.04_Na_0.04_Mn^2+^_0.03_Al_0.03_Cr^3+^_0.01_)_Σ=1.00_Si_1.00_O_3_. At the last refinement stage, with anisotropic atomic displacement parameters for all atoms and no constraints, the residual value settled at *R*_1_(*F*) = 0.0593 for 187 observed reflections [*F*_o_ > 4σ(*F*_o_)] and 17 parameters and at *R*_1_(*F*) = 0.0892 for all 270 independent reflections.

Crystallographic data (CCDC 1522131) can be obtained free of charge from *The Cambridge Crystallographic Data Centre* via www.ccdc.cam.ac.uk/data_request/cif.

### X-ray powder diffraction

X-ray powder diffraction data (Table S4) were obtained with an Oxford Diffraction Xcalibur PX Ultra diffractometer fitted with a 165 mm diagonal Onyx CCD detector and using copper radiation (Cu*K*α, λ = 1.54138 Å). The working conditions were 50 kV × 50 nA with 7 hours of exposure; the detector-to-sample distance was 7 cm. The program *Crysalis* RED^56^ was used to convert the observed diffraction rings to a conventional powder diffraction pattern. The least squares refinement gave the following unit-cell values: *a* = 4.7490(2), *c* = 13.6934(9) Å, *V* = 267.45(2) Å^3^.

## Additional Information

**How to cite this article**: Bindi, L. *et al*. Discovery of the Fe-analogue of akimotoite in the shocked Suizhou L6 chondrite. *Sci. Rep.* **7**, 42674; doi: 10.1038/srep42674 (2017).

**Publisher's note:** Springer Nature remains neutral with regard to jurisdictional claims in published maps and institutional affiliations.

## Supplementary Material

Supplementary Information

## Figures and Tables

**Table 1:** Electron microprobe analyses (means and ranges in wt% of oxides) of hemleyite, olivine and pyroxene of the Suizhou meteorite.

	hemleyite		olivine		clinoenstatite		Fe-rich clinoenstatite	
	mean	ranges	mean	ranges	mean	ranges	mean	ranges
SiO_2_	51.08	50.68–51.85	40.97	39.55–41.16	56.42	55.98–57.09	52.48	52.18–53.01
Al_2_O_3_	1.26	1.11–2.55	0.00	0.00–0.04	0.21	0.13–0.39	1.68	1.42–1.99
Cr_2_O_3_	0.61	0.29–1.25	0.01	0.00–0.05	0.15	0.09–0.31	3.13	2.88–3.45
FeO	29.33	26.88–30.52	8.78	8.24–9.15	13.68	13.02–14.25	25.25	24.90–25.66
MgO	12.71	11.21–13.10	49.27	48.95–49.66	27.96	27.22–28.75	13.46	13.08–14.02
CaO	1.88	0.95–2.03	0.09	0.03–0.14	0.82	0.45–1.11	0.66	0.55–0.80
MnO	1.76	1.44–2.05	0.01	0.00–0.04	0.50	0.31–0.86	1.22	0.88–1.37
Na_2_O	1.02	0.88–1.39	0.00	0.00–0.02	0.08	0.03–0.21	2.17	1.52–2.60
Total	99.65	98.87–100.38	99.11	98.89–99.93	99.82	99.06–100.29	100.05	99.47–100.70
Si	1.00		1.01		1.01		1.01	
Al	0.03		0.00		0.00		0.04	
Cr^3+^	0.01		0.00		0.00		0.05	
Fe^2+^	0.48		0.18		0.20		0.40	
Mg	0.37		1.80		0.75		0.38	
Ca	0.04		0.00		0.02		0.01	
Mn^2+^	0.03		0.00		0.01		0.02	
Na	0.04		0.00		0.00		0.08	
Σcat	2.00		2.99		1.99		1.99	

Chemical formulae were calculated on the basis of three (hemleyite and pyroxene) and four (olivine) oxygen atoms.

Fe and Mn were assumed as divalent.
