# Supplementary material for: Discovery of the Fe-analogue of akimotoite in the shocked Suizhou L6 chondrite
Source: Sci Rep. 2017 Feb 15;7:42674. doi: 10.1038/srep42674 (PMC5309820; doi:10.1038/srep42674)
Supplement: Supplementary Information [file srep42674-s1.pdf]

SUPPLEMENTARY INFORMATION

Discovery of the Fe-analogue of akimotoite in the shocked Suizhou L6 chondrite

Luca Bindi[1,2,*], Ming Chen[3,4] , Xiande Xie[4,5]

[1]Dipartimento di Scienze della Terra, Università di Firenze, Via La Pira 4, I-50121 Florence, Italy

[2]CNR-Istituto di Geoscienze e Greorisorse, Via La Pira 4, I-50121 Florence, Italy

[3]State Key Laboratory of Isotope Geochemistry, Guangzhou Institute of Geochemistry, Chinese Academy of Sciences, Guangzhou 510640, China

[4]Guangdong Provincial Key Laboratory of Mineral Physics and Materials, Guangzhou 510640, China

[5]Key Laboratory of Mineralogy and Metallogeny, Guangzhou Institute of Geochemistry, Chinese Academy of Sciences, Guangzhou 510640, China

*Corresponding Author: luca.bindi@unifi.it

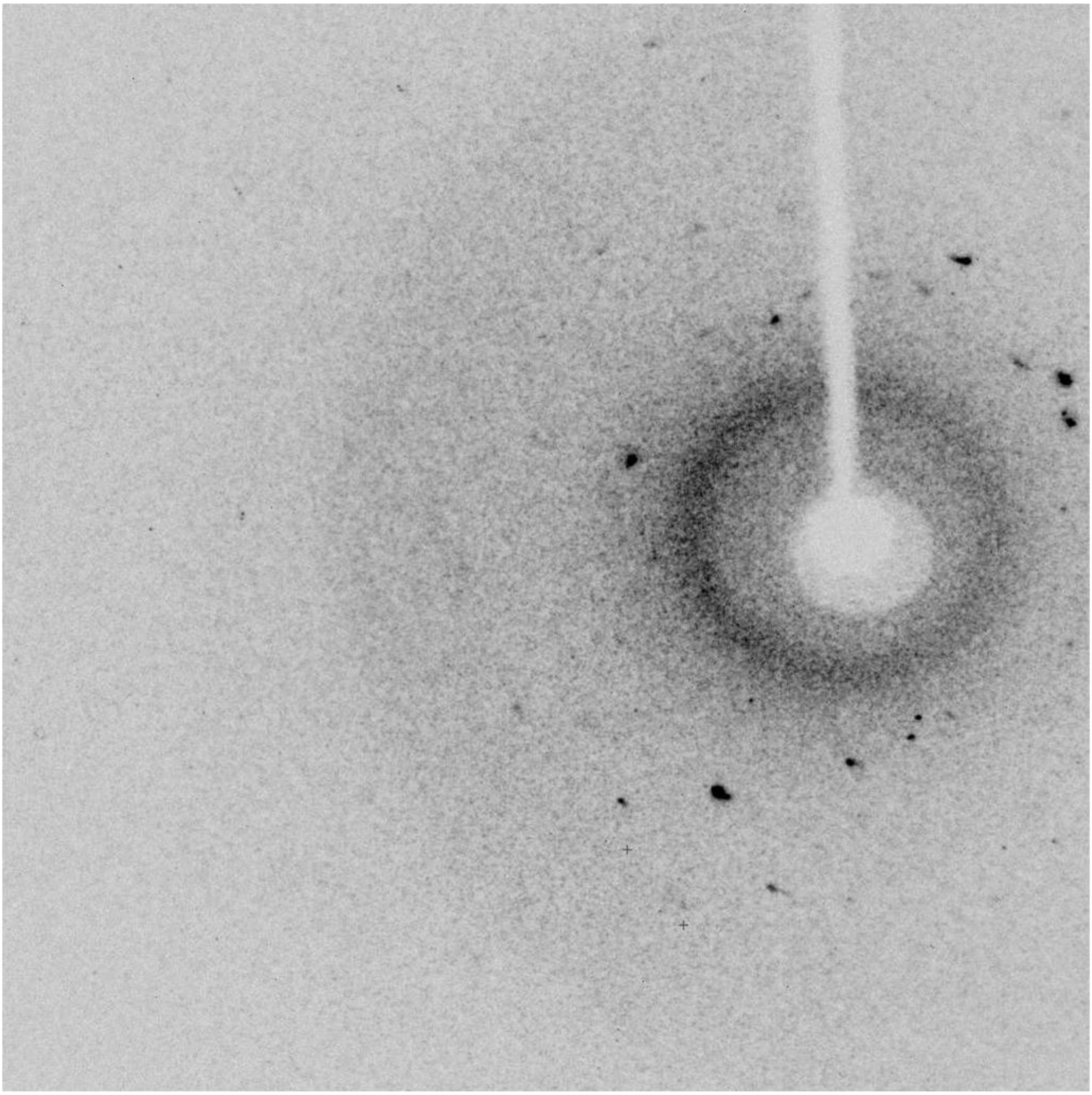

Figure S1. X-ray frame collected with the single-crystal diffractometer. Diffraction spots belong to hemleyite whereas diffraction rings belong to polycrystalline pyroxene.

Table S1. Wyckoff positions, atom coordinates and equivalent isotropic displacement parameters (Å^2) for hemleyite.

atom	*Wyckoff*	*x*/*a*	*y*/*b*	*z*/*c*	U_{iso}
A (Fe,Mg)	6*c*	0	0	0.3585(2)	0.0227(9)
B (Si)	6*c*	0	0	0.1567(2)	0.0207(9)
O	18*f*	0.326(1)	0.298(1)	0.2351(3)	0.027(1)

Table S2. Anisotropic displacement parameters (Å^2) for hemleyite.

atom	U_{11}	U_{22}	U_{33}	U_{12}	U_{13}	U_{23}
A (Fe,Mg)	0.0222(11)	0.0222(11)	0.0236(13)	0.0111(5)	0	0
B (Si)	0.0214(12)	0.0214(12)	0.0193(14)	0.0107(6)	0	0
O	0.024(3)	0.027(3)	0.029(2)	0.012(2)	-0.0005(18)	0.0000(19)

Table S3. Selected bond distances (Å) for hemleyite.

A-	O	2.000(5)	(×3)	B-	O	1.734(5)	(×3)
	O	2.249(5)	(×3)		O	1.833(5)	(×3)
	mean	2.13			mean	1.78	

Table S4. Observed and calculated X-ray powder diffraction data (d in Å) for hemleyite. The strongest reflections are given in bold.

			1		2	
h	k	l	d_{obs}	I_{est}	d_{calc}	I_{calc}
1	0	1	-	-	3.9377	5
**0**	**1**	**2**	**3.520**	**35**	**3.5233**	**41**
**1**	**0**	**4**	**2.625**	**100**	**2.6277**	**100**
**1**	**1**	**0**	**2.376**	**50**	**2.3742**	**53**
0	1	5	-	-	2.2761	5
**2**	**-1**	**3**	**2.105**	**50**	**2.1053**	**43**
1	1	3			2.1053	5
0	2	1	-	-	2.0332	6
1	0	7	-	-	1.7635	5
0	2	4	1.762	25	1.7616	31
2	-1	6	**1.645**	**50**	1.6435	16
**1**	**1**	**6**			**1.6435**	**46**
0	1	8	-	-	1.5774	6
3	-1	4	-	-	1.4147	9
2	1	4	1.415	10	1.4147	17
3	0	0	1.372	20	1.3707	33
1	0	10	1.300	5	1.2968	10
2	-1	9	-	-	1.2791	6
2	2	0	-	-	1.1871	6
0	2	10	-	-	1.1381	7
-1	4	4	-	-	1.0818	6
4	-2	6	-	-	1.0527	7
3	-1	10	-	-	1.0263	6

1 = observed diffraction pattern; 2 = calculated diffraction pattern obtained with the atom coordinates reported in Table S1 (only reflections with $I_{rel} \geq 4$ are listed).
